# Effects of Multiple Stressors, Pristine or Sulfidized Silver Nanomaterials, and a Pathogen on a Model Soil Nematode *Caenorhabditis elegans*

**DOI:** 10.3390/nano14110913

**Published:** 2024-05-23

**Authors:** Jarad P. Cochran, Phocheng Ngy, Jason M. Unrine, Christopher J. Matocha, Olga V. Tsyusko

**Affiliations:** 1 Department of Plant and Soil Sciences, College of Agriculture, Food and Environment, University of Kentucky, Lexington, KY 40546, USA; jaradcochran@uky.edu (J.P.C.); jason.unrine@uky.edu (J.M.U.); cjmato2@uky.edu (C.J.M.); 2 Kentucky Water Research Institute, University of Kentucky, Lexington, KY 40506, USA

**Keywords:** biofilm, internal colonization, nanotoxicity, toxicity, antagonistic effects

## Abstract

Previous research using the model soil nematode *Caenorhabditis elegans* has revealed that silver nanoparticles (AgNP) and their transformed counterpart, sulfidized AgNP (sAgNP), reduce their reproduction and survival. To expand our understanding of the environmental consequences of released NP, we examined the synergistic/antagonistic effects of AgNP and sAgNP along with AgNO_3_ (ionic control) on *C. elegans* infected with the pathogen *Klebsiella pneumoniae*. Individual exposures to each stressor significantly decreased nematode reproduction compared to controls. Combined exposures to equitoxic EC_30_ concentrations of two stressors, Ag in nanoparticulate (AgNP or sAgNP) or ionic form and the pathogen *K. pneumoniae*, showed a decline in the reproduction that was not significantly different compared to individual exposures of each of the stressors. The lack of enhanced toxicity after simultaneous combined exposure is partially due to Ag decreasing *K. pneumoniae* pathogenicity by inhibiting biofilm production outside the nematode and significantly reducing viable pathogens inside the host. Taken together, our results indicate that by hindering the ability of *K. pneumoniae* to colonize the nematode’s intestine, Ag reduces *K. pneumoniae* pathogenicity regardless of Ag form. These results differ from our previous research where simultaneous exposure to zinc oxide (ZnO) NP and *K. pneumoniae* led to a reproduction level that was not significantly different from the controls.

## 1. Introduction

Silver nanoparticles (AgNP) are currently one of the most extensively employed nanoparticles because of their distinctive antibacterial and antifungal properties [1,2]. Silver NP are found in clothes, water filters, antifouling membranes, medical devices, biosensors, and nano-pesticides [3,4,5]. From these products, some AgNP are released into wastewater, which eventually arrives at wastewater treatment plants (WWTP) [6,7] and is subsequently applied to agricultural soils via the application of contaminated biosolids [8]. In the case of Ag-based nano-pesticides, NP are directly released into the environment through their application to agricultural fields. As AgNP enter WWTP or the environment, they are subjected to a biogeochemically driven transformation process [9]. The transformation of these NP is dependent upon pH, dissolved oxygen content, and the ligands AgNP interacts with but the primary transformation is from zerovalent AgNP to sulfidized AgNP (sAgNP) [10]. This transformation alters AgNP solubility and subsequently their toxicity [11,12]. Typically, sulfidation greatly reduces the toxicity of AgNP [13], partially due to diminished oxidative stress, which has been shown to be induced by increased ROS production after exposure to pristine but not sulfidized AgNP [14]. However, sAgNP exposure has been shown to decrease the biomass of soil organisms [15] and high concentrations still exert toxic effects, with concentrations of sAgNP 250 µg/L and above resulting in increased nematode mortality [12]. Additionally, although sAgNP is stable in most environments, sAgNP dissolves in iron-rich acidic soils, which leads to leaching of toxic Ag^+^ ions [16]. Even as undissolved NP, long-term exposure to sAgNP has been shown to elicit the stress response in bacteria [17] and in *C. elegans,* multigenerational exposure leads to increased sensitivity to Ag toxicity [18]. As such, to predict the environmental risk of released AgNP, it is necessary to assess the toxicity of both pristine AgNP and its environmentally transformed counterpart, sAgNP.

The few studies examining AgNP and sAgNP toxicity at smaller environmentally relevant concentrations and conditions show that they exhibit negative effects on model soil organisms [12,19,20]. For most nanomaterial toxicity experiments, model organisms are subjected to a single stressor, such as NP. While experiments with a single stressor are crucial for elucidating the consequences of released NP, in the environment, organisms contend with multiple stressors, such as pathogens. There is a noticeable scarcity of research examining the combined impacts of these stressors. In environments characterized by multiple stressors, the exposure of organisms to NP may result in different responses compared to single-species laboratory exposures, driven by synergistic or antagonistic interactions between NP toxicity and biotic factors [21,22]. In the context of infection within a host, the additional stress from the pathogen might intensify or potentially mitigate the adverse effects of NP exposure [23]. While interspecies interactions have been explored for some NP [24,25], to our knowledge, no study has yet examined the effects of AgNP toxicity on a host infected with a pathogen at the same time. Such investigations play a crucial role in comprehending the ecological relevance of released AgNP and the potential effects they may trigger.

Therefore, we examined the effects of exposing *Caenorhabditis elegans*, a well-established model organism in toxicology and environmental studies, to AgNO_3_, AgNP, and sAgNP, alongside a common gram-negative pathogen, *Klebsiella pneumoniae*. The soil-dwelling nematode *C. elegans* offers a unique opportunity to unravel the intricate dynamics between NP and pathogens [26,27,28]. The nematode presents several advantages, including its short generation time, abundant reproduction, and low maintenance cost, allowing for relatively swift and comprehensive experiments [28,29]. Nanomaterial toxicity studies of AgNP on *C. elegans* have shown decreased reproduction and lifespan with increased mortality after exposure [30,31]. The transformed product, sAgNP, has also been shown to cause these negative effects but requires larger concentrations [12]. The nematode is also susceptible to many common pathogens, such as *K. pneumoniae*, an often-studied pathogen in human and animal health [32]. This pathogen has been shown to cause mortality after 48 h of infection [33] and if *C. elegans* is infected during its reproductive window, it decreases the nematode’s reproductive output [23].

This study is a continuation of our research on the effects of NP exposure in the context of multiple stressors [23]. Our previous research focused on zinc oxide (ZnO) NP, which showed that ZnONP attenuates much of *K. pneumoniae* pathogenicity in *C. elegans* without exerting a toxic effect on nematodes, even though the concentration tested was above therapeutic levels. However, Zn, being an essential nutrient, has specific molecular pathways in *C. elegans* to maintain homeostasis [34]. Because Ag has no biological function in the nematode, the effects of AgNP might differ compared to ZnONP. Thus, to better understand the environmental repercussions of released NP, this study investigated the combined effects after *C. elegans* exposure to AgNP (and sAgNP) and *K. pneumoniae*. Specifically, the objectives were to assess whether *C. elegans* responses to the two combined stressors, AgNP (or sAgNP) and *K. pneumoniae*, are significantly altered compared to the effects of each individual stressor. The ionic Ag control was also included in all individual and combined exposures and its EC_30_ is within the range for the predicted environmental concentrations for agricultural soils that receive biosolids [35]. Furthermore, the study aimed to investigate whether AgNP (or sAgNP) inhibits *K. pneumoniae* biofilm formation and its internal colonization both prior to and after NP has been ingested by *C. elegans*. Our hypothesis was that the simultaneous exposure of *C. elegans* to both stressors, AgNP (or sAgNP) and *K. pneumoniae*, would enhance toxicity compared to pathogen exposure alone, due to the heightened stress from NP exposure.

## 2. Materials and Methods

### 2.1. Nanoparticles, Sulfidation, and Characterization

Polyvinylpyrrolidone (PVP) coated AgNP (50 nm, 1 mg/mL, AGPB50-1M) were purchased from nanoComposix, Inc. (San Diego, CA, USA), and used as pristine AgNP. For sulfidized sAgNP treatment, the pristine AgNP was sulfidized using a method outlined in Levard et al. [11]. Briefly, AgNP were combined with Na_2_S at a 1:2 molar ratio of Ag to S. For this synthesis, while vortexing a 500 µL solution of 9.2 mM AgNP, 500 µL of 18.4 mM Na_2_S reaction solution was added. The tubes were left uncapped for 4 h, sealed, and left undisturbed for 4 days. The particles were then separated from the Na_2_S solution by centrifugation at 14,000 rpm for 30 min, washed twice with 18 MΩ deionized water (DI H_2_O), and resuspended in 500 µL DI H_2_O. The mean intensity hydrodynamic diameter (z-average) was measured via dynamic light scattering (DLS, Malvern ZetaSizer Nano-ZS, Malvern, UK) using the exposure media at 100 ng Ag/mL. Hückel approximation from electrophoretic mobilities, which was measured with phase analysis light scattering (PALS, Malvern Zetasizer Nano-ZS), was used to estimate the ζ-potential of the particles. To validate both AgNP and sAgNP sizes, 10 µL of 100 NP µg/mL was deposited onto lacey carbon films using copper grids. Particle size and energy dispersive X-ray spectroscopy (EDS) were assessed with a Talos F200X transmission electron microscope (TEM, Thermo Fisher Scientific, Waltham, MA, USA). To determine the primary particle size distribution, the diameters of approximately 100 individual particles from three separate images were measured using ImageJ ver. 1.54b software (https://imagej.nih.gov/ij/).

To further characterize the crystal structure of the AgNP and sAgNP, thin films of pristine AgNP, sAgNP, and an authentic Ag_2_S standard (99.999% pure, Sigma-Aldrich, St. Louis, MO, USA) were deposited onto glass petrographic slides. Powder X-ray diffraction patterns were collected with a Malvern PANalytical X’Pert Pro X-ray diffractometer using CuKα radiation (Westborough, MA, USA). Additionally, Selective Area (Electron) Diffraction (SAED) and Fast-Fourier Transform (FFT) patterns of sAgNP were also acquired with the Talos F200X TEM (Thermo Fisher Scientific, Waltham, MA, USA). The patterns were analyzed with the ImageJ add-on, Fiji ver. 2.15.1 (https://imagej.net/software/fiji/).

Previous research has shown that AgNP toxicity can largely be attributed to Ag^+^ ion release [36]. Therefore, the dissolutions of AgNP and sAgNP in our exposure solutions were assessed. After setting up exposure experiments outlined below, an aliquot (1 mL) was taken from each treatment, centrifuged at 14,000 rpm for 30 min, and the supernatant removed, which represents the Ag concentration at the initial stage. After 24 h, an additional aliquot was taken, representing Ag concentrations at the end of the experiment. To determine if AgNP dissolution was caused by bacterial biomass or the ligands produced by the microbes, experimental solutions without Ag were incubated for 24 h. Next, the tubes were centrifuged, the supernatant removed, and Ag was added like in a typical experiment and incubated for another 24 h. Each dissolution experiment was conducted in duplicate. These samples were then digested following EPA method 3005A [37], which has been validated for recovery of Ag. Whole suspensions and supernatants were acid-digested prior to ICP-MS analysis. Briefly, samples were acidified to 9% HNO_3_ and 3% HCl and then digested in a CEM Mars 6 microwave digestion system by heating to 180 C and holding that temperature for 15 min in sealed Teflon digestion vessels (Matthews, NC, USA). Digestates were diluted, internal standard was added, and Ag concentrations were measured and analyzed via ICP-MS Inductively Coupled Plasma Mass Spectrometry (ICP-MS; Agilent 7900, Santa Clara, CA, USA). Quality control parameters followed the U.S. EPA method 6020B [38] and included method blanks, initial and ongoing blank and calibration verification, duplicates, and spike recovery. Unless otherwise stated, after each experiment, Ag concentrations in exposure solutions were measured using this method.

### 2.2. Strains, Nematode Maintenance, and Exposure Conditions

*Klebsiella pneumoniae* (ATCC 10231) was obtained from the American Type Culture Collection (ATCC, Manassas, VA, USA). Prior to each experiment, a glycerol stock with *K. pneumoniae* was thawed out and Luria-Bertani broth (LB; 0.5% yeast extract, 1.0% tryptone, and 0.5% NaCl) was added. After adding ampicillin at 100 µg/mL, the *K. pneumoniae* stock was placed into an incubator at 37 °C for 24 h. Afterward, to remove LB broth, the stock was centrifuged at 14,000 rpm for 10 min, washed, and resuspended with a low-ionic medium Environmental Protection Agency moderately hard reconstituted water (MHRW) [39]. The bacterial concentration was then calculated from the optical density at 600 nm (OD_600_).

*Caenorhabditis elegans* wild-type N2 was sourced from the Caenorhabditis Genetic Center (CGC, Minneapolis, MN, USA). Unless otherwise stated, nematodes were fed an *Escherichia coli* OP50. Because *E. coli* OP50 is unable to synthesize uracil, a nucleotide essential for RNA production, it is a slow-growing bacterium and unable to produce a biofilm, rendering it non-pathogenic [40]. To ensure that there is a sufficient amount of food for the nematodes during toxicity experiments, OP50 is grown prior to the exposures. Additionally, previous research shows that dead bacteria are a viable food source for nematodes [41]. Therefore, the toxic effects of Ag on *E. coli* OP50 should not reduce food availability. Previously developed protocols were followed for nematode maintenance and age-synchronization [42]. The age-synchronization was achieved using a NaClO/NaOH solution to isolate the nematode eggs, which were then cultivated at 20 °C on K-agar plates containing *E. coli* OP50 bacterial lawn. Unless specified otherwise, all exposures were carried out in MHRW supplemented with 5 µL glucose (20%) per mL of MHRW. For each experiment, age-synchronized nematodes at the L3 developmental stage were distributed among seven treatment groups: *E. coli* OP50, Ag NP:: *E. coli* OP50, AgNP:: *K. pneumoniae*, AgNO_3_:: *E. coli* OP50, AgNO_3_:: *K. pneumoniae*, sAgNP:: *E. coli* OP50, and sAgNP:: *K. pneumoniae*.

### 2.3. Evaluating Effects on Nematode Reproduction

To evaluate antagonistic/synergistic effects in response to pathogen and AgNP stress, *C. elegans* reproduction was measured following exposure to multiple stressors, AgNP and/or *K. pneumoniae*. Previously, we examined different toxicity endpoints for *C. elegans* responses to AgNP and sAgNP [12] and a more sensitive reproduction endpoint was selected for this study to evaluate the effect of the combined stressors. Additionally, our previous findings have indicated that exposure to NP at EC50 levels results in higher mortality rates compared to EC30, which might skew the results [12]. Therefore, EC30 values of AgNP, sAgNP, and AgNO_3_ (for an ionic control) on nematode reproduction were used. The respective EC_30_ values of AgNO_3_, AgNP, and sAgNP were 11 µg/L, 275 µg/L, and 2200 µg/L (Figure S3). To begin the experiment, age-synchronized nematodes at the L3 stage (around 100) were exposed to Ag, along with either non-pathogenic *E. coli* OP50 or pathogenic *K. pneumoniae* for 24 h. After that exposure, six nematodes from each group were transferred to individual 3-cm K-agar plates containing *E. coli* OP50. The plates were left at 20 °C in the dark for 48 h to allow the nematodes to lay their eggs. After 48 h, the original nematode was transferred to a fresh K-agar plate and incubated for an additional 24 h. Subsequently, after a total of 72 h, Rose Bengal (0.5 mg/L) was added to the plates for staining and they were heated at 50 °C for 55 min. Fully hatched offspring were counted under a Leica S6D digital stereo microscope (Leica Microsystems, Wetzlar, Germany).

### 2.4. Nematode Growth Assay

Each group of nematodes was incubated within individual 15 mL centrifuge tubes, containing 4 mL of the exposure solutions as described above, with each Ag concentration at their respective EC_30_ for reproduction. Nematodes were subjected to an incubation period of 24 h at 20 °C. Following this incubation, a minimum of 20 nematodes were selected from each exposure group. These nematodes were immobilized using sodium azide (3 μL of 170 mM) and imaged through light microscopy. The images obtained were then analyzed with ImageJ software (https://imagej.nih.gov/ij/) to quantify the surface area of the nematodes’ two-dimensional projection.

### 2.5. Biofilm Formation Assay

Quantitative analysis of biofilm growth was accomplished following a modified method from Liu, et al. [43]. To begin, 5 mL of each exposure solution was aliquoted into separate 6-cm sterile Petri dishes, with three dishes for each treatment. The cultures were then statically incubated at 37 °C for 24 h before being discarded. Afterward, each dish underwent a triple wash with phosphate-buffered saline (PBS) followed by staining with 0.1% crystal violet in ethanol for 20 min. After the staining, the plates were washed thrice with deionized (DI) water and were left to air dry overnight. To measure absorbance, the absorbed dye was solubilized using 5 mL of ethanol. Next, 200 µL samples from each plate were pipetted into a 96-well plate and the absorbance was measured at 570 nm.

### 2.6. Quantification of Internal Pathogens

To determine the colonization of *K. pneumoniae* in the nematodes’ intestine, a modified method detailed by Kamaladevi and Balamurugan [33] was followed. After an 8 h exposure period, the 15-mL tubes with the nematodes were centrifuged for 1 min at 1000× *g* and the supernatant was removed. The nematodes then underwent two washes with MHRW, after which they were transferred to 4 mL of MHRW supplemented with *E. coli* OP50, to remove any unadhered *K. pneumoniae* in the intestines. This mixture was left to incubate for 16 h. To prevent peristalsis, the nematodes were chilled before the next series of washes. Following this, nematodes were washed twice with 0.1% Triton X-100 in MHRW and rinsed once more with MHRW. For each treatment, we selected 10 individual nematodes that were ground within PVP microcentrifuge. The lysates obtained were added to LB agar plates containing 100 μg/mL ampicillin and incubated at 37 °C for 24 h. The colony-forming units (CFUs) were counted the subsequent day.

### 2.7. Statistical Analysis

For normally distributed data, one-way analysis of variance (ANOVA) followed by a Dunnett’s test or Student’s *t*-test was used to test for statistically significant differences (i.e., *p* ≤ 0.05) when compared to controls. For non-normally distributed data (i.e., growth data), the Mann–Whitney U test was used to compare each treatment to the control. Q-Q plots and the Shapiro–Wilk test were used to check for normal distributions of variance. The EC_30_ values for Ag ions, AgNP, and sAgNP were calculated from linear regression parameters. Statistical analyses were performed in R (4.1.2)

## 3. Results and Discussion

The main finding of this study is that the simultaneous exposure of Ag, whether as ionic, particulate, or sulfidized forms, with the pathogen *K. pneumoniae* led to neither antagonistic nor synergistic negative effects. In other words, the biological indicators selected for assessing negative effects on our model species, *C. elegans*, were not significantly different when exposed to multiple stressors versus single stressors. Our findings suggest that though Ag mitigates *K. pneumoniae* pathogenicity by reducing the bacteria’s ability to colonize its host, the toxic effect of Ag is still exerted on the nematodes. This result is the opposite of findings in our recent publication, Cochran et al. (2023) [23], where it was shown that Zn attenuates much of *K. pneumoniae* pathogenicity in *C. elegans* without exerting a toxic effect on nematodes, despite being at a concentration above therapeutic levels [23]. We explore more of a comprehensive discussion of our findings below.

### 3.1. Particle Characterization and Dissolution

**Figure 1:**
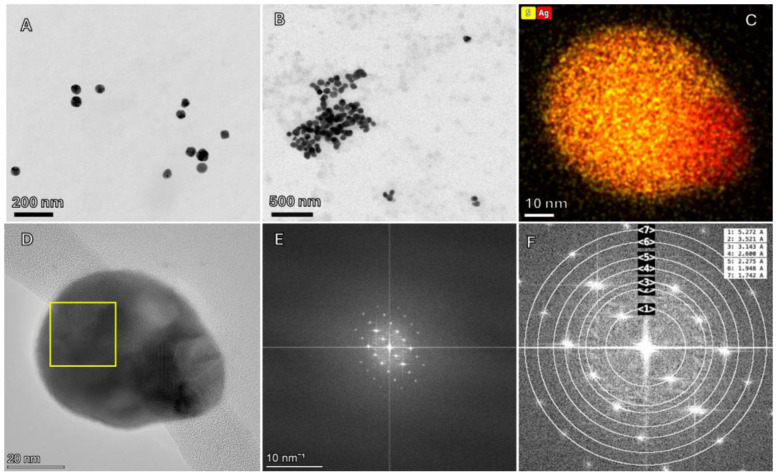
Transmission electron microscopy images (TEM) of (**A**) AgNP and (**B**) sAgNP. Energy dispersive spectroscopy (**C**) shows that sAgNP is composed of S and Ag; (**D**) High-resolution TEM of sAgNP with the selected area indicated by the yellow box; (**E**) Fast Fourier transform-pattern (FFT) of inset in panel (**D**); (**F**) Fitting of FFT pattern and calculated interplanar (d) spacings in Å (d) = 3.53 (3.57), 3.14 (3.08), 2.60 (2.61), 2.28 (2.21), 1.94 (1.94), and 1.74 (1.73), indicating acanthite structured Ag_2_S. The distances in parenthesis are the previously reported values from the International Center for Diffraction Data (ICDD) file 14–72. FFT was performed using Fiji software. The TEM plug-in for imageJ was used to fit FFT data and calculate d-spacings.

AgNP and sAgNP had a primary particle size ranging from 15–60 nm and 32–81 nm, respectively, with a median size of 35 nm (SD = 8 nm) for AgNP (Figure 1A) and 54 nm (SD = 10 nm) for sAgNP (Figure 1B). The pristine NP was spherical in shape but the sulfidation altered the NP into a hexagonal shape. The elemental maps clearly demonstrate the sulfidation of the AgNP as indicated by the co-occurrence of S and Ag (Figure 1C). Some areas of the particles were more enriched in Ag than others, suggesting the presence of some unreacted elemental Ag. However, powder XRD analysis demonstrated an almost complete disappearance of the face-centered cubic elemental Ag peaks at 2.36 and 2.06 Å indicating nearly complete sulfidation of the pristine AgNP (Figure S1). This is in agreement with the previous study by Levard et al. [11] who demonstrated that the method we used results in complete sulfidation of Ag through both powder XRD and extended X-ray absorption fine structure (EXAFS) spectroscopy. These enriched Ag areas in the elemental maps may therefore be the result of photo-reduction from the electron beam. The absence of defined acanthite peaks, as observed in the authentic Ag_2_S standard, in the sAgNP, demonstrates that the Ag_2_S in the particles is amorphous and lacking in long-range crystal structure which would diffract X-rays. It has been previously observed that the majority of formed Ag_2_S is amorphous at all ratios of Ag:S studied and was therefore undetectable by XRD [11]. Indeed, the weak diffraction shown in the SAED pattern of a sAgNP shows that the particle is poorly crystalline, likely due to a lack of a long-range order (Figure S2). The FFT pattern and derived d-spacings show that the particle is composed of acanthite-structured Ag_2_S (Figure 1D,E).

**Figure 2:**
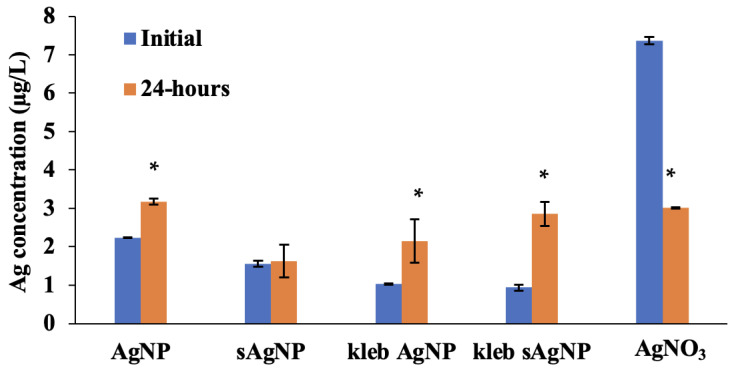
Ag concentration in supernatants of *C. elegans* exposure media at the beginning of the experiment and after 24 h. The concentrations of AgNO_3_, AgNP, and sAgNP added to the exposure solutions corresponded to their respective EC_30_ (i.e., 11 µg/L, 275 µg/L, and 2200 µg/L). Data are presented with error bars representing ± 1 SD. The asterisk indicates significant differences in Ag^+^ concentrations initially and after 24 h (*p* < 0.05).

A volume-weighted hydrodynamic diameter of AgNP in MHRW at pH 7.2 was 28.6 nm with a ζ-potential of −12.6 mV (SD = 11.3 mV). Sulfidized sAgNP had a hydrodynamic diameter of 32.8 nm with a ζ-potential of 0.0 mV (SD = 7.1 mV). The zeta potential of sAgNP has changed because of the PVP coating removal during sulfidation and the sulfidized particle is now made from Ag_2_S and not Ag. The hydrodynamic diameter has increased because of the sulfur addition to the structure. The dissolution experiments, which were conducted in the presence of *C. elegans* and bacteria (*E. coli* OP50 or *K. pneumoniae*), showed little dissolution from either NP, regardless of the type of bacteria present (Figure 2). Compared to initial concentrations of AgNP and sAgNP (i.e., 275 ng/mL and 2200 ng/mL, respectively), 99.3% and 99.9% of Ag from AgNP and sAgNP were recovered from the resulting pellet after centrifuging the solutions. Interestingly, AgNP and sAgNP showed a greater dissolution in the presence of *K. pneumoniae* than with *E. coli* OP50. This is likely due to the higher metabolic rate of *K. pneumoniae* compared to the slow-growing *E. coli* OP50. Numerous studies have shown that metabolically active bacteria produce extracellular polysaccharides in the presence of Ag [44,45,46,47,48]. The S moieties on these biomolecules, such as thiols (R-SH), have a high affinity to Ag [49] and can potentially bind to the surface of these NPs, altering their dissolution rates [50]. Finally, in the case of AgNO_3_, centrifugation should not reduce Ag concentration in the supernatant because it is ionic Ag^+^. However, Ag^+^ bound to cell surfaces would be strongly bound to sulfhydryl groups and this explains why the concentration of free ionic Ag from the AgNO_3_ treatment decreased significantly over the incubation period after 24 h in the presence of biomass. This could be due to the reduction of Ag^+^ to Ag^0^ in the microbial suspension or binding of Ag^+^ to microbial cells and nematodes or uptake into these organisms as the organisms would be removed from the supernatant by the centrifugation step. Indeed, when comparing the free ionic Ag concentrations of the AgNO_3_ treatments in both the experiments without bacteria and bacteria removed, a lesser variation in concentrations between the initial and 24 h time points is observed (Figure S4A), suggesting that the bacteria are needed for the Ag^+^ to precipitate. Reduction in Ag on the surface of the bacterial cell wall has been shown to occur with *K. pneumoniae* [51]. Because of this, the concentration of free ionic Ag when at equilibrium in the exposure solution (e.g., not bound to organisms or reduced to elemental Ag) is likely reflected by the final Ag concentration from the AgNO_3_ treatment, i.e., 3 µg/L.

### 3.2. Silver Reduces Klebsiella pneumoniae Pathogenicity but Still Exerts Toxicity

**Figure 3:**
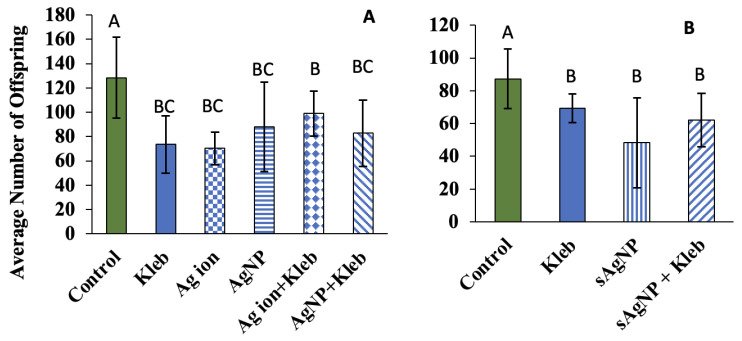
Average total number of offspring per adult *C. elegans* after 24 h exposure to (**A**) Ag ions and AgNP or (**B**) sAgNP at EC_30_ concentrations (11 µg/L, 275 µg/L, and 2500 µg/L, respectively) with *E. coli* OP50 or *K. pneumoniae* in moderately hard reconstituted water (±1 SD). Significant differences among treatments (*p* < 0.05) are indicated with letters (i.e., A, B, or C). Treatments sharing the same letter are not significantly different from each other.

Exposure of *C. elegans* at EC_30_ to AgNO_3_, AgNP, or sAgNP for 24 h significantly decreased reproduction compared to controls (Figure 3). When exposed to *K. pneumoniae* with or without Ag, reproduction was also repressed. The results differ from Kim et al. (2018) [25] who found that AgNP toxicity was largely avoided by pre-infecting *C. elegans* with *Pseudomonas aeruginosa*, another commonly studied gram-negative pathogen [25]. Their research showed that pre-infection upregulated the PMK-1/p38 MAPK pathway, a highly conserved molecular pathway related to innate immunity and stress responses [33].

The difference between the two, however, is likely due to the timing, conditions, and AgNP concentrations of the exposure, where the nematodes were pre-infected for eight hours, allowed to recover for six hours, and exposed to AgNP at LC_50_ without feeding. In our previous toxicogenomic study with AgNP, despite differences in the experimental conditions, we observed similar increases in the expression of such stress-response genes, such as *numr-1*, *gst-4*, *gst-20*, and *lys-2* as well as activation of the lysozyme pathway in ionic Ag treatment [52]. However, in this study, during combined simultaneous exposure of *C. elegans* to both stressors, *K. pneumoniae* and AgNP, these responses, even if they stayed the same, were not sufficient to reduce toxicity. Additionally, in the combined exposure scenario, *K. pneumoniae* is also being exposed to AgNP, which disrupts *K. pneumoniae* biofilm and extracellular polymeric substances production. By hindering the pathogen’s ability to avoid the host immune response, AgNP is effectively reducing its pathogenicity.

**Figure 4:**
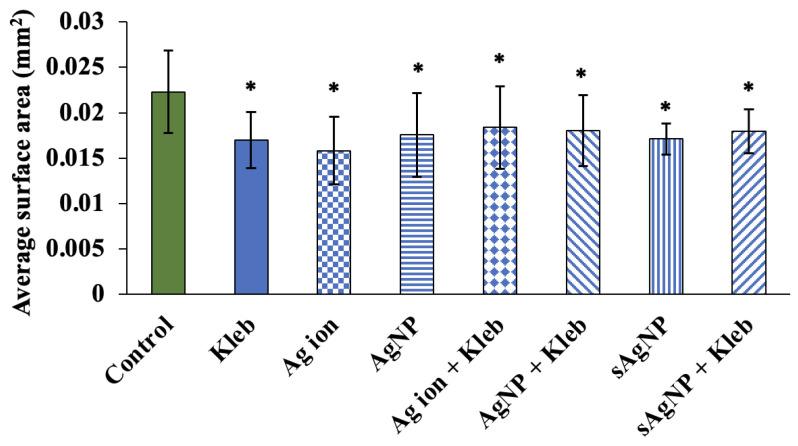
Average surface area of nematodes exposed to Ag ions, AgNP, or sAgNP at EC_30_ concentrations with *E. coli* OP50 or *K. pneumoniae* in moderately hard reconstituted water after 24 h (±1 SD, N = 20). Treatments that are significantly different from the control are shown with an asterisk based on the Mann–Whitney U test (*p* < 0.05).

Based on Mann–Whitney U tests, comparison of each treatment to the control showed significant decreases in growth, as measured by surface area, after exposure to AgNO_3_, AgNP, and sAgNP (*p* < 0.05; Figure 4). Additionally, exposure to *K. pneumoniae*, without Ag and with Ag, also significantly decreased growth (Figure 4). The mean body area for the controls was 0.022 mm^2^. The greatest difference between the control mean area was observed in AgNO_3_ exposure (0.016 mm^2^). The smallest difference was observed in AgNO_3_ with *K. pneumoniae* (0.018 mm^2^). Also, neither reproduction nor growth was synergistically or additively decreased when combined with multiple stressors. This again points to the negative effects of AgNP on *K. pneumoniae*. In other words, the decrease in biological endpoints to assess *C. elegans* health can be attributed mostly to Ag exposure, regardless of its form.

### 3.3. Ag Ions, AgNP, and sAgNP Reduce Klebsiella pneumoniae Colonization

**Figure 5:**
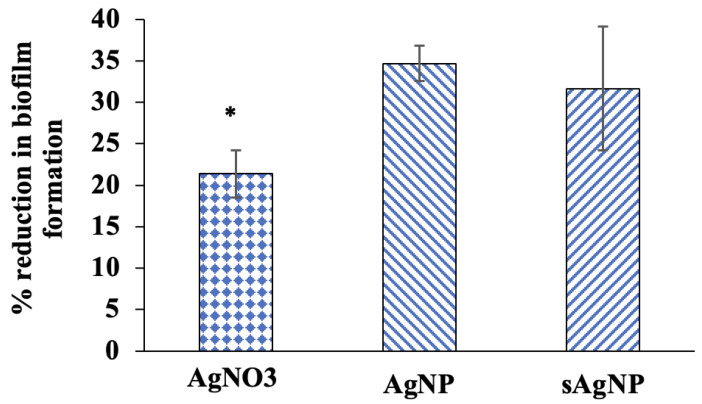
Biofilm inhibition of *K. pneumoniae* by AgNO_3_, AgNP, or sAgNP at EC_30_ concentrations over 24 h (±1 SD). All treatments reduced *K. pneumoniae* biofilm formation. The % reduction was measured relative to the absorbance calculated from *K. pneumoniae* treatment without exposure, which was used as the baseline for biofilm formation. Asterisks show significant differences among treatments (*p* < 0.05).

**Figure 6:**
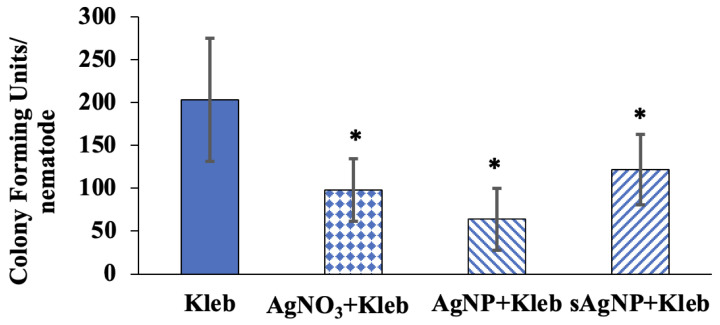
Colony-forming units per nematode after *C. elegans* were fed *K. pneumoniae* with or without AgNO_3_, AgNP, or sAgNP at EC_30_ concentrations for 24 h (±1 SD, N = 8). The asterisks designate treatments that are significantly different from the control (*p* < 0.05).

To assess the effects of Ag exposure on *K. pneumoniae*’s ability to infect *C. elegans*, the pathogen’s ability to produce biofilms outside the host and its viability inside the nematode was determined. Quantitative assessments showed that AgNO_3_, AgNP, and sAgNP decreased biofilm formation (Figure 5). While these evaluations do not precisely mimic the intricate in vivo conditions within the *C. elegans* intestine, they provide evidence that Ag adversely affects *K. pneumoniae*’s ability to form biofilms, potentially reducing the pathogen’s colonization in the host intestine. Indeed, our assessment of Ag effects on viable pathogens within infected nematodes showed significant decreases in CFU, which represents living bacteria in the host (Figure 6).

In this experiment, nematodes were fed *K. pneumoniae* for 8 h and after that transferred to the plates with *E. coli* OP50 for 16 h. The mean CFU of nematodes fed on *K. pneumoniae* was 203 CFU per nematode. AgNO_3_-exposed nematodes contained an average of 98 CFU, whereas AgNP reduced viable bacteria to 64 CFU. The sAgNP treatment had the smallest reduction in viable bacteria with 122 CFU. Taken together, the biofilm production assay and assessment of viable bacteria inside *C. elegans* indicate that Ag negatively affects *K. pneumoniae*, before and after ingestion, hampering its ability to successfully infect its host. Specifically, by disrupting the pathogen’s ability to form a biofilm, an essential requirement to attach to a host’s intestine and avoid its immune response [53,54], Ag thwarts the pathogen from colonizing *C. elegans*, reducing its pathogenicity.

## 4. Conclusions

These results are similar to our previous findings where ZnONP was shown to inhibit *K. pneumoniae* biofilm production and reduce its pathogenicity in *C. elegans*. However, simultaneous exposure of *C. elegans* to both *K. pneumoniae* and ZnONP resulted in a reversal of negative effects from both stressors, i.e., reproduction returned to levels comparable to controls [23]. It remains to be determined why *K. pneumoniae* exposure coincided with a reduction in ZnONP toxicity but not AgNP toxicity. When comparing the two NP, it is important to consider the biological functions (or lack thereof) of its composition, i.e., Ag versus Zn. Silver has no biological function inside *C. elegans*, whereas Zn is a cofactor in multiple enzymes [34,55]. As such, nematodes have effective mechanisms in maintaining Zn homeostasis [56]. However, it is unknown if these mechanisms might be used during pathogen infection. Therefore, more research on *C. elegans*’ ability to maintain homeostasis in the presence of pathogens is needed.

Due to the increased use of AgNP, it is becoming inevitable that soil organisms will encounter AgNP and sAgNP alongside various other stressors. To better predict the effect of multiple stressors on these organisms, this study explored the interactions between AgNP and pathogens to ascertain potential antagonistic/synergistic effects. The differing results between this study and our previous study with ZnONP show the complex interactions between NP and organisms. Delving deeper into these interactions and their underlying mechanisms may offer insights into the intricate interplay between organisms’ innate immunity and toxicant defenses. Further research could illuminate how these interactions ultimately impact population-level dynamics in the environment.

## Data Availability

Data are available on request.

## Supplementary Materials

The following supporting information can be downloaded at https://www.mdpi.com/article/10.3390/nano14110913/s1: Figure S1: The X-ray diffraction patterns of silver sulfide (Ag_2_S), silver nanoparticles (AgNP), and sulfidized silver nanoparticles (sAgNP).; Figure S2: The selected area diffraction (SAED) pattern of a sulfidized silver nanoparticle (sAgNP). Figure S3: Mean total number of offspring produced per adult *Caenorhabditis elegans* exposure to (A) AgNO_3_, (B) Ag NP, or (C) sAg NP in MHRW in the presence of food *E. coli* OP50; Figure S4: Ag concentration in supernatants of *C. elegans* exposure media at the beginning of the experiment and after 24 h. Mean total number of offspring produced per adult *Caenorhabditis elegans* after its exposure to (A) AgNO_3_, (B) Ag NP, or (C) sAg NP in MHRW in the presence of food *E. coli* OP50 (±1 SD).
