# Supplementary material for: Effects of Multiple Stressors, Pristine or Sulfidized Silver Nanomaterials, and a Pathogen on a Model Soil Nematode Caenorhabditis elegans
Source: Nanomaterials (Basel). 2024 May 23;14(11):913. doi: 10.3390/nano14110913 (PMC11173860; doi:10.3390/nano14110913)
Supplement: Supplementary file 1 [file nanomaterials-14-00913-s001.zip › nanomaterials-2995947-supplementary.pdf]

Supplementary Information

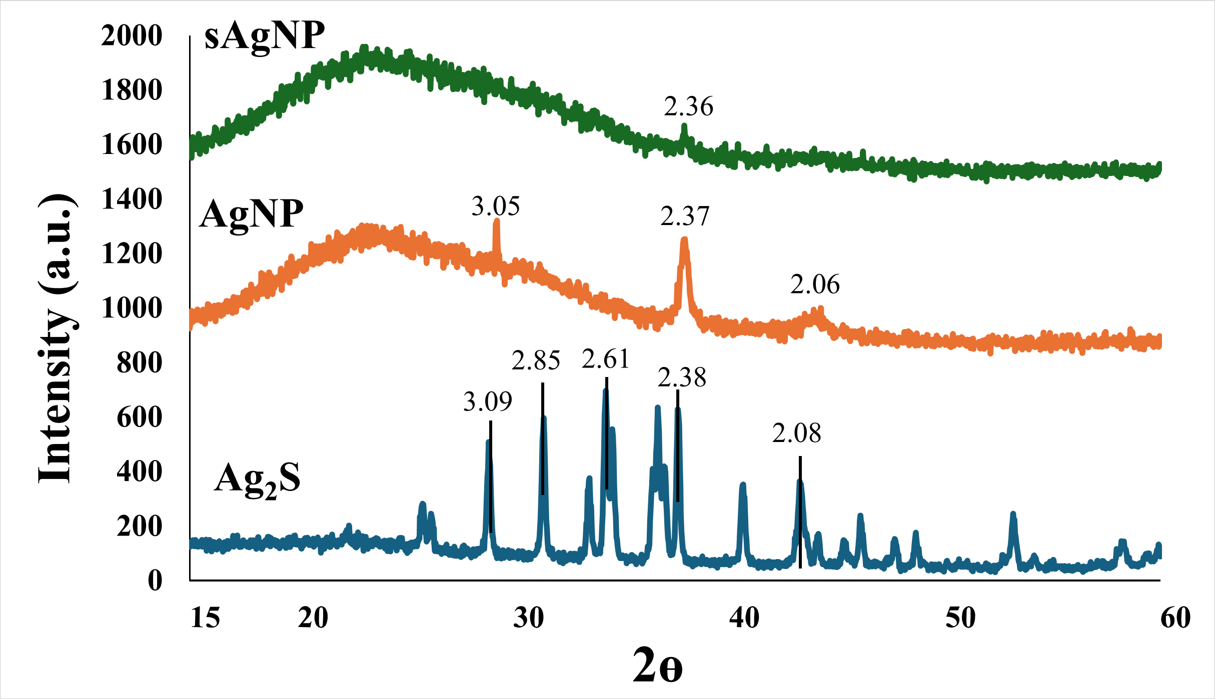

Figure S1. The X-ray diffraction patterns of silver sulfide (Ag_2S), silver nanoparticles (AgNP), and sulfidized silver nanoparticles (sAgNP). Numbers on the graph indicate the d-spacing [Å] for the corresponding peak. Intensity is shown as arbitrary units.

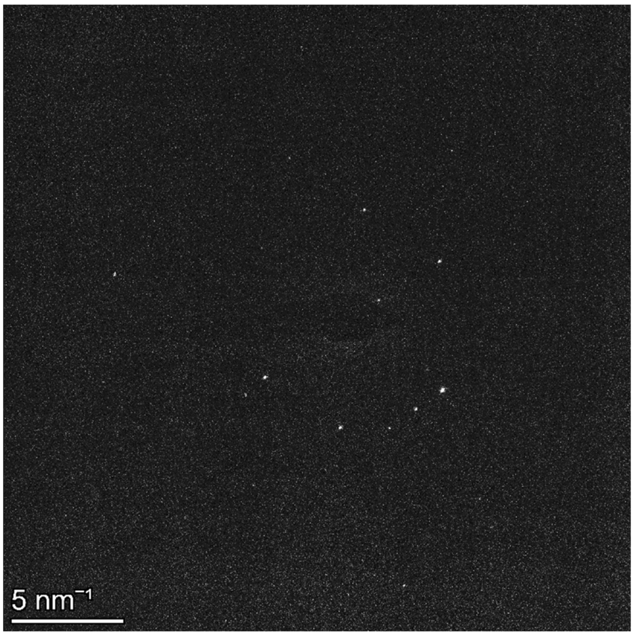

Figure S2. The selected area diffraction (SAED) pattern of a sulfidized silver nanoparticle (sAgNP).

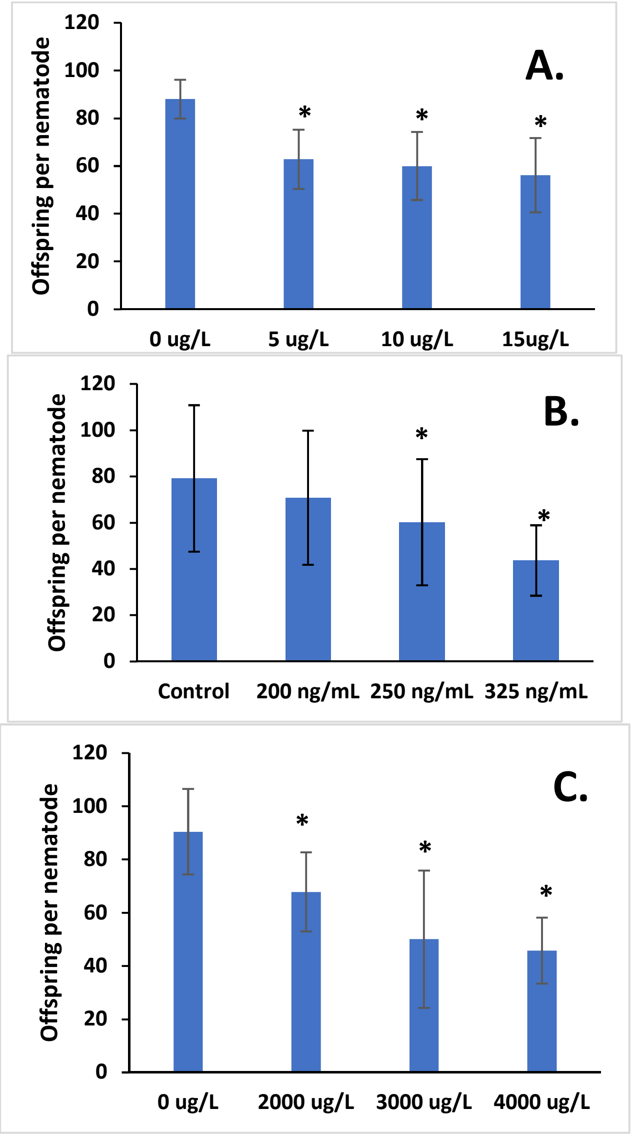

Figure S3. Mean total number of offspring produced per adult *Caenorhabditis elegans* exposure to (A) $AgNO_3$, (B) Ag NP, or (C) sAg NP in MHRW in the presence of food *E. coli* OP50 (± 1 SD). * indicates concentrations significantly different compared to the controls.

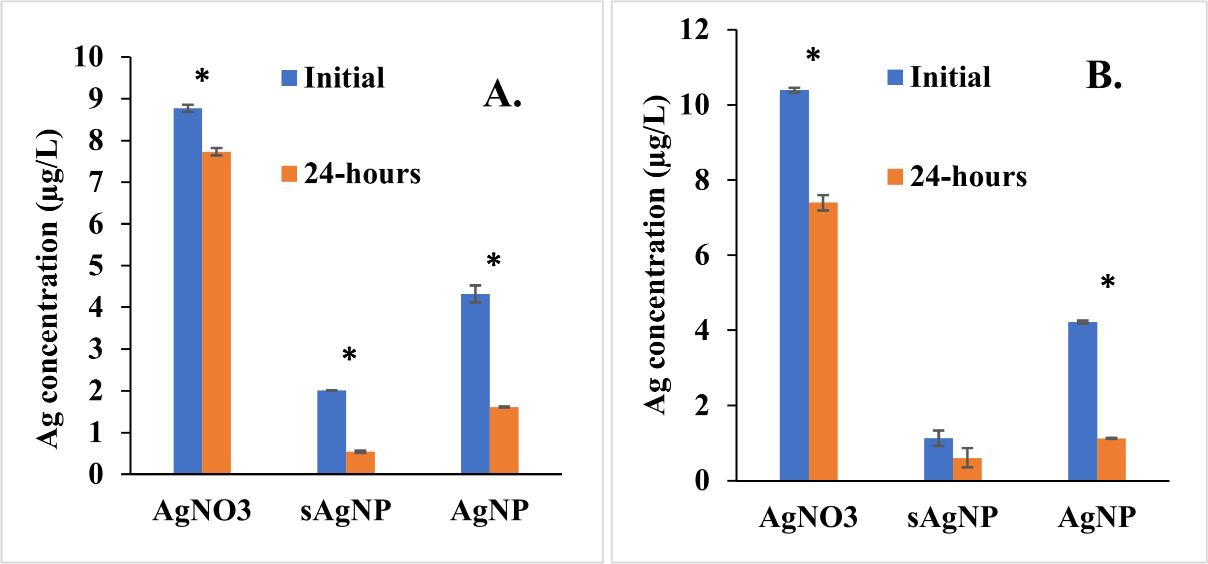

Figure S4. Ag concentration in supernatants of *C. elegans* exposure media at the beginning of the experiment and after 24 h. Graphs show concentrations (A) without bacteria and (B) with bacteria grown for 24 h and then removed. The concentrations of $AgNO_3$, AgNP, and sAgNP added to the exposure solutions corresponded to their respective EC_{30} (i.e., 11 µg/L, 275 µg/L, and 2200 µg/L). Data are presented with error bars representing ± 1 SD. Treatments with significantly different Ag concentrations after 24 h are shown with an asterisk ($p < 0.05$). Each experiment was conducted in duplicate for all treatments.
